# Supplementary material for: Creep-type all-solid-state cathode achieving long life
Source: Nat Commun. 2024 May 2;15:3706. doi: 10.1038/s41467-024-48174-8 (PMC11065878; doi:10.1038/s41467-024-48174-8)
Supplement: Supplementary file 1 — Supplementary Information [file 41467_2024_48174_MOESM1_ESM.pdf]

Supplementary Information for

Creep-type All-solid-state Cathode Achieving Long Life

Xiaolin Xiong[1,2], Ting Lin[1], Chunxi Tian[1,2], Guoliang Jiang[1,2], Rong Xu[3]*, Hong Li[1], Liquan Chen[1], Liumin Suo[1,2]*

Table of Contents

Section 1. Supplementary Text 2

Section 2. Screening for creepable cathode materials 4

Section 3. Construct creep-type all-solid-state cathodes 7

Section 4. In situ observation of creep behavior of Se 18

Section 5. Comparison for mechanical properties on creep behavior of materials ..26

Section 6. Electrochemical performance of creep-type Se-60MSe cathode. 39

References 48

[1]Beijing National Laboratory for Condensed Matter Physics, Institute of Physics, Chinese Academy of Science; Beijing, 100190, China

[2]Center of Materials Science and Optoelectronics Engineering, University of Chinese Academy of Sciences; Beijing, 100049, China

[3]State Key Lab for Strength and Vibration of Mechanical Structures, Department of Engineering Mechanics, Xi'an Jiaotong University; Xi'an, 710049, China

*e-mail: suoliumin@iphy.ac.cn; rongxu@xjtu.edu.cn

Section 1. Supplementary Text

Calculation Details of density and porosity

$$\rho_C = \frac{m_C}{V_C} = \frac{m_C}{S \cdot h_C} \quad (1)$$

$$\varphi_C = \frac{V_C - V_0}{V_C} = \left(1 - \frac{\rho_C}{\rho_0}\right) \cdot 100\% \quad (2)$$

ρ_C is the actual density of the cathode by measuring, m_C, V_C, h_C and φ_C represents the mass, volume, height the porosity of the cathode, respectively. V_0 and ρ_0 is the theoretical volume and density of cathode material.

$$\rho_0 = \frac{1}{\frac{x}{\rho_a} + \frac{y}{\rho_b}} \quad (3)$$

x and y are the mass ratios of Se and MSe in Se-MSe composite cathode, for different proportions, Se-MSe would also be written as Se-yMSe. ρ_a and ρ_b are the theoretical density of Se (4.81 g/cm^3) and MSe (6.52 g/cm^3). All determined densities and porosities are susceptible to errors, as high as 10%, due to the inaccurate determination of composite cathode thickness.

Calculation of Energy density.

The calculation model of the energy density of the Se electrode was according to the previous works by Erik J. Berg[1] and Meiying Li[2]. For the Liquid Li-ion batteries (LLIBs), the cathode consists of 60 wt.% active material (AM, Se, 4.81 g/cm^3) and 30 wt.% conductive carbon (Super P (SP), 2.25 g/cm^3) and 10 wt.% binder (1.8 g/cm^3) with 30 vol.% porosity filled with liquid electrolyte (1M $LiPF_6$ in EC: EMC, 1.22 g/cm^3). The cathode of traditional all-solid-state batteries (ASSBs) contains 40 wt.% AM and 50 wt.% SE (Li_6PS_5Cl, 1.64 g/cm^3) and 10 wt.% SP [2], i.e. 40 wt.% Se +50 wt.% SE +10 wt.% SP (Se-50SE-10SP). The all-electrochemical-active (AEA) all-solid-state Se-MSe cathodes in this work only contain Se and Mo_6Se_8 two phases in different proportions without SE and SP. The theoretical specific capacity of Se is 675 mAh/g and Mo_6Se_8 is 89 mAh/g which exhibits a stable capacity of 80 mAh/g in ASSBs (Fig. S3). Porosity is obtained by measuring the thickness of the electrode at different pressures (Table. S4, S5). Theoretical densities come from Material Project Database.

The density of cathode in LLIBs:

$$\rho_{cathode} = \left(\frac{1}{\frac{0.6}{\rho_{Se}} + \frac{0.3}{\rho_{CC}} + \frac{0.1}{\rho_{Binder}}}\right) \cdot (1 - \varphi) + \rho_{LE} \cdot \varphi \quad (4)$$

The density of cathode in traditional ASSBs:

$$\rho_{cathode} = \frac{1}{\frac{0.4}{\rho_{Se}} + \frac{0.5}{\rho_{SSE}} + \frac{0.1}{\rho_{CC}}} \cdot (1 - \varphi) \quad (5)$$

The density of Se-MSe cathode:

$$\rho_{Se-MSe} = \frac{1}{\frac{x}{\rho_{Se}}+\frac{y}{\rho_{MSe}}} \cdot (1-\varphi) \tag{6}$$

Specific capacity of cathode in LIBs and SSBs:

$$C_{cathode} = \frac{C_{Se} \cdot m_{se}}{m_{cathode}} \tag{7}$$

Specific capacity of Se-MSe cathode:

$$C_{cathode} = \frac{C_{Se} \cdot m_{se} + C_{MSe} \cdot m_{Mse}}{m_{cathode}} \tag{8}$$

Specific energy of cathode:

$$E_G = \int C_{cathode}\, dV = C_{cathode} \cdot V_{average} \;\; (Wh/kg) \tag{9}$$

Volumetric energy density of cathode:

$$E_V = \frac{C_{cathode} \cdot V_{average} \cdot m_{cathode}}{V_0} \;(Wh/L) \tag{10}$$

$V_{average}$ is the average voltage of material during discharge.

Active utilization of Se in Se-MSe cathode:

$$\varepsilon = \frac{C_{cathode} - C_{MSe} \cdot y}{C_{Se} \cdot x} \cdot 100\% \tag{11}$$

Supplementary Note

The hardness of lithiated InLi alloy (E~46 GPa, H~1.8 GPa, T_m~600 °C) increase with increasing Li content[3]. And it should be noted that InLi alloy is brittle at low temperature without viscosity below about 200°C[4]. Furthermore, in contrast with many other intermetallic compounds, LiIn alloy don't experience the defect softening phenomenon even at high homologous temperatures even at temperatures up to 450 °C (~0.8 T_m), even though Indium (E=12.7 GPa, T_m=156.6 °C) is similar to Li and prone to creep and stress relaxation[5].

In addition, even though the practical ASSBs might eventually use Li foil as the anode, the creep of Li foil only improves the contact condition between the Li and SSE, while not affects the microstructural evolution within the cathode.

Section 2. Screening for creepable cathode materials

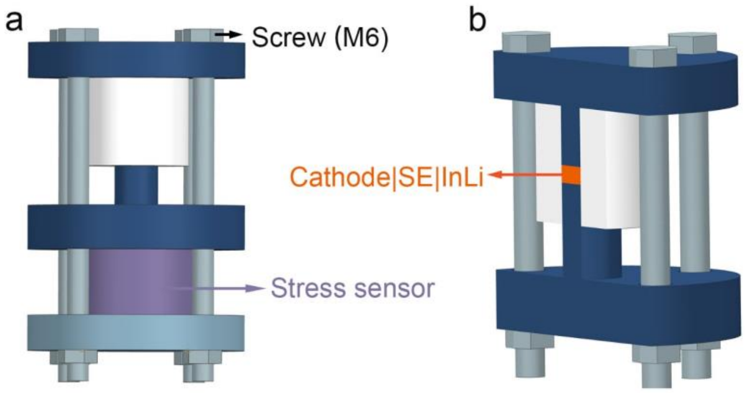

Fig. S1 | Schematic diagram of ASSBs mold. (a) Initial stack stress measurement device with stress sensors. (b) Cross-section of the ASSBs test setup**.**

For this stainless-steel mold, the torque force of the screw translates into stack pressure on the ASSBs. The initial stacking pressure exerted by an M6 screw gram is about 100MPa (~8 kN) tested by stress sensor. It is worth noting that shear stress may vary with the applied external because the microscopic forces on individual particles are anisotropic and depend on the local stress environment. Currently, it is difficult to measure internal microscopic stresses inside ASSBs experimentally, since prior computational simulations have demonstrated that shear stress in particles tends to be higher than external stack stress[6,7].

Table S1. Physical properties of common electrode materials.

Materials	Shear Modulus (GPa)	Melting Point T_M (K)	$T_{298\,K}/T_M$	$T_{343\,K}/T_M$	Ref.
LCO	80	1373	0.22	0.25	[8]
NCM111	78	1373	0.22	0.25	[8]
LMO	77	1273	0.23	0.27	[8]
LFPO	45.5	1273	0.23	0.27	[9]
S	6.82	385.8	0.77	0.89	[10]
Se	3.7	493.8	0.60	0.69	[10]
Si	62	1683	0.18	0.20	[11]
Sn	18	504.9	0.59	0.68	[12]
Ga	18	302.8	0.98	1.13	[12]
Ge	41	1211.2	0.25	0.28	[11]
Te	16	722.5	0.41	0.47	[10]
Sb	20	903.6	0.33	0.38	[13]
In	4.4	429.6	0.69	0.80	[12]

The mechanical data and melting points without reference of these materials are obtained from the Material Project website.

Table S2. Electrochemical and structural properties of common electrode materials.

Material	Capacity (mAh/g)	Voltage (V)	Density (g/cm^3)	Volumetric capacity (mAh/cm^3)	Relative volume change (%)	Ref.
**LCO**	274	3.9	4.97	1363	2	14,15
**NCM111**	280	3.9	4.76	1333	2	14,15
**LMO**	148	4.1	4.25	629	5.6	15,16
**LFPO**	170	3.4	3.6	612	7	15
**Li_2S**	1166	2.28	1.66	1936	79	15
**Li_2Se**	578	2.07	2.87	1658.86	98	15
**$Li_{15}Si_4$**	1857	0.4	0.63	1167	270	17
**$Li_{22}Sn_4$**	790	0.38	2.58	2038	255	15
**Li_2Ga**	641	0.6	2.98	1910	160	18
**$L_{22}Ge_4$**	1143	0.4	2.04	2332	240	15
**Li_2Te**	379	1.86	3.4	1288	104.7	15,19
**Li_3Sb**	564	0.87	2.98	1680	135	20
**$Li_{13}In_3$**	801	0.2	2.47	1978	134	21
**Li**	3860	0	0.534	2061	∞	
**$Li_4Mo_6Se_8$**	89	2.2	6.52	580.3	11.2	22

The homogenous temperature T_H indicates that, under some stress circumstances, both S and Se can creep at room temperature. Given Se and S have comparable volumetric capacity (3253 mA h/cm^3 for Se and 3467 mA h/cm^3 for S), but compared to S (5×10^{-28} S/cm) [23], Se has a significantly higher electrical conductivity (10^{-3} S/cm) [24], which means that Se can occupy a much higher active content in the electrode than S, indicating that Se as the active material promises superior electrochemical performance for all-solid-state cathode.

Section 3. Construct creep-type all-solid-state cathodes

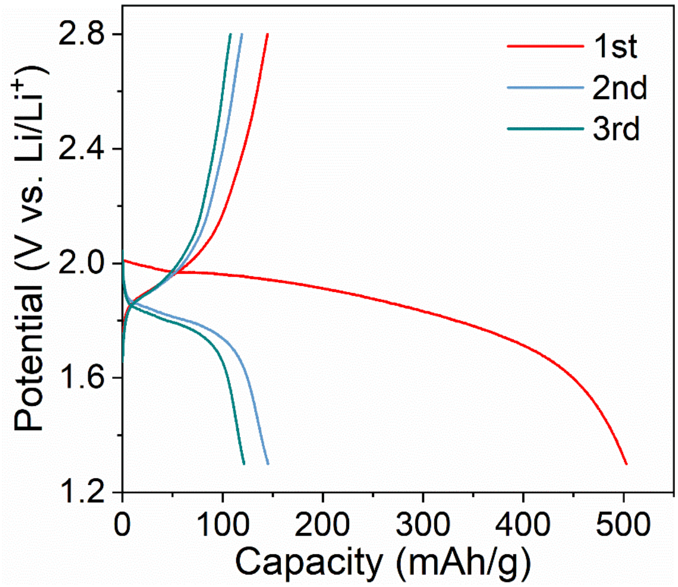

Fig. S2 | The galvanostatic charge-discharge curves of Se-50SE-10SP composite cathodes.

Large volume changes of Se during charging and discharging process in conventional all-solid-state selenium electrodes without structural design may end up in electrochemical capacity loss owing to particles contact failure.

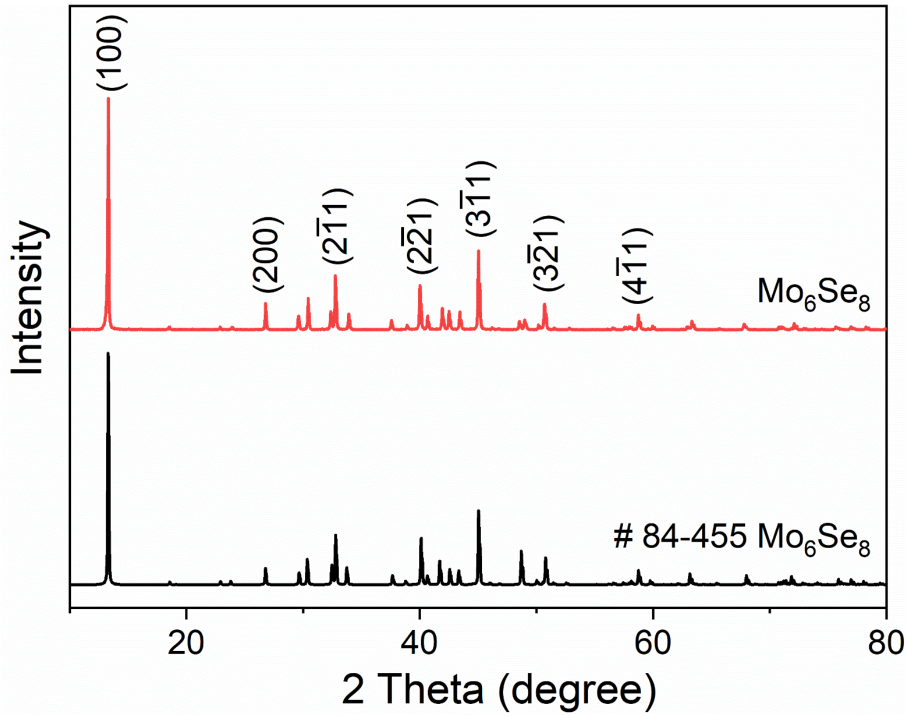

Fig. S3 | The XRD patterns of the Mo_6Se_8 sample.

106 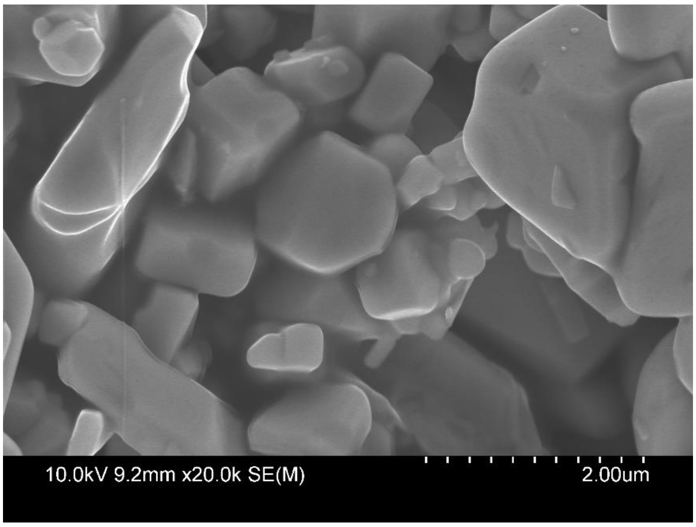

107 **Fig. S4 |** SEM image of Mo_6Se_8.

108

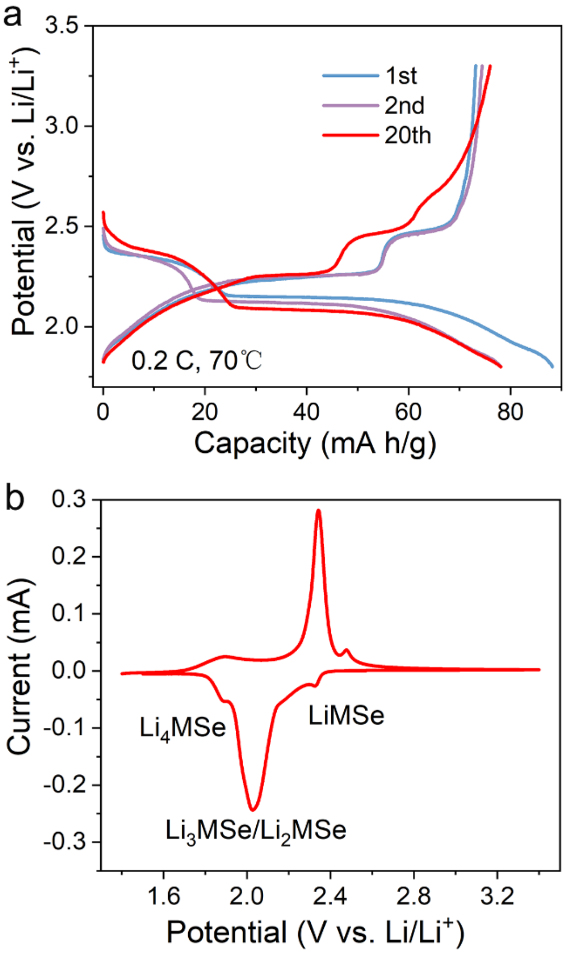

Fig. S5 | Electrochemical performance of Mo_6Se_8 in ASSBs. (a) The galvanostatic charge-discharge curves of Mo_6Se_8 for the 1st, 2nd, and 20th cycles. (b) Cyclic voltammetry of Mo_6Se_8 electrode between 1.4-3.4 V at a scan rate of 0.5 mV/s.

114

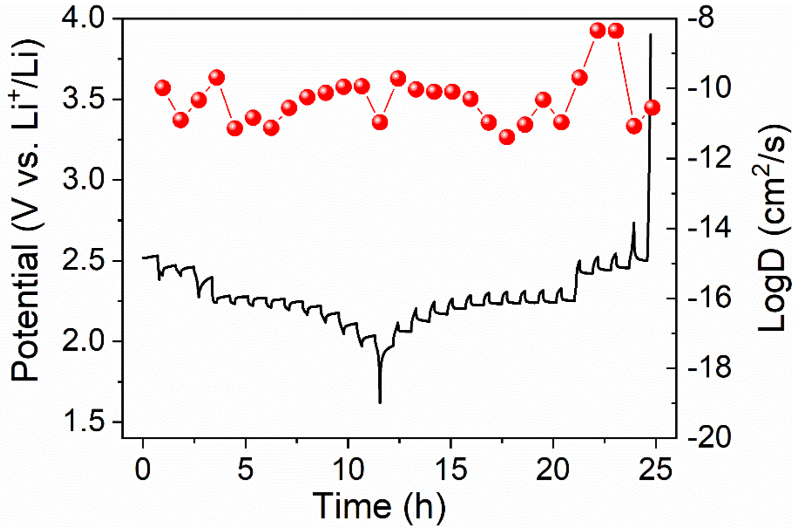

115 **Fig. S6 |** The Li-ion diffusion coefficients of Mo_6Se_8 were obtained from GITT.

116

Table S3. Details of electronic conductivity measurements of Mo_6Se_8 and Super P using four-pin probe method under different pressure.

Material	Pressure [MPa]	Thickness [mm]	Conductivity [S/cm]	Density [g/cm^3]
**Mo_6Se_8**	9.55	1.87	32.45	3.44
	19.10	1.78	59.62	3.61
	28.65	1.74	85.32	3.70
	38.20	1.71	110.72	3.76
	47.75	1.68	132.91	3.85
	57.30	1.65	154.18	3.90
**Super P**	9.55	4.84	12.95	0.71
	19.10	4.21	18.80	0.82
	28.65	4.86	23.57	0.89
	38.20	3.62	27.69	0.95
	47.75	3.43	31.45	1.00
	57.30	3.29	34.77	1.05

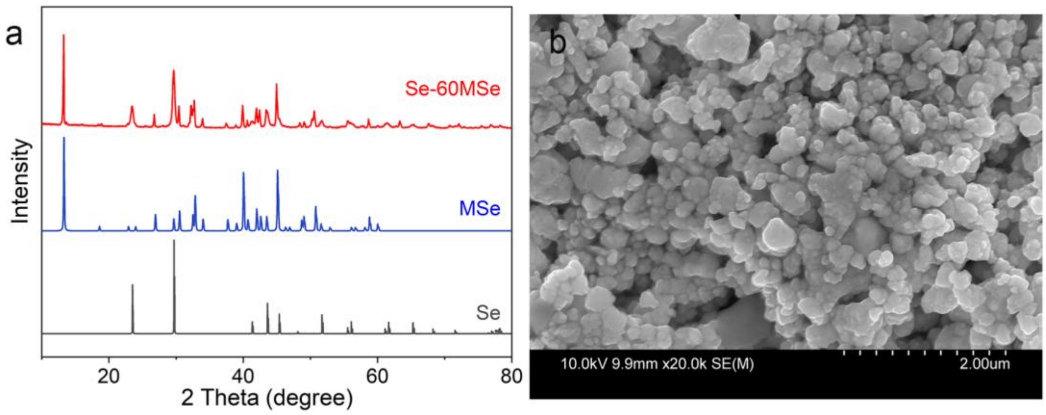

Fig. S7 | (a) The XRD patterns and (b) SEM image of Se-60MSe powder.

As illustrated by the XRD patterns, there are no new phases generated after the ball-milling mixing process, which suggests no reaction between Se and MSe, and the constructed Se-60MSe cathode remains a stable phase structure.

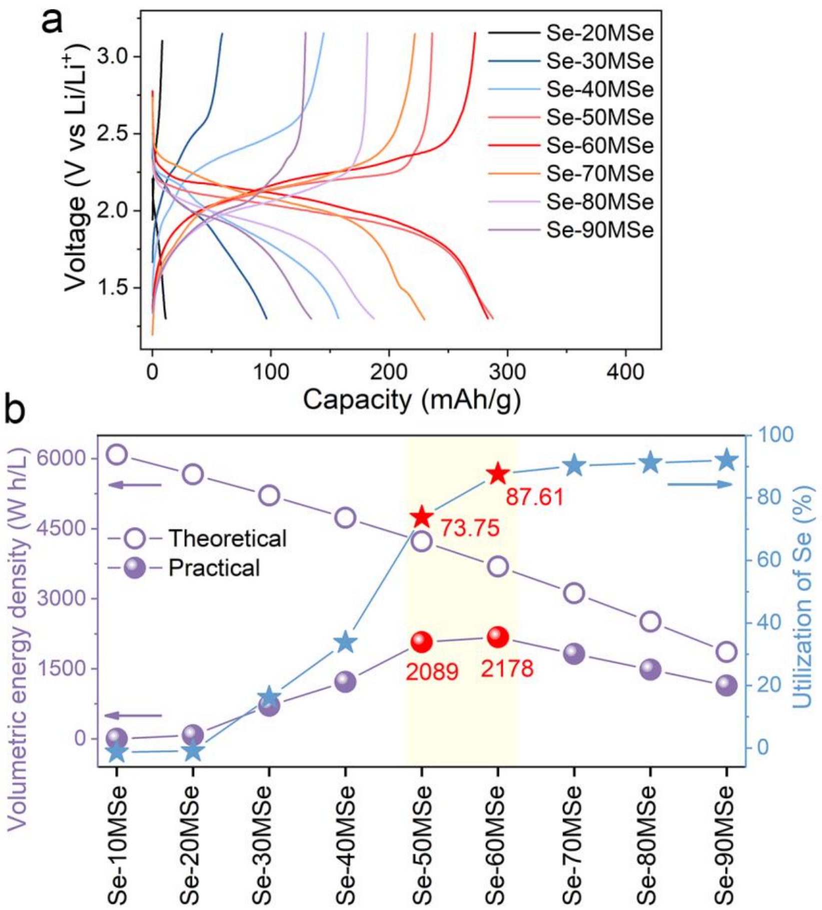

Fig. S8 | Comparison of electrochemical performance of Se-MSe with different mixing ratios. (a) The galvanostatic charge-discharge curves of Se-MSe composite electrodes for the 2nd cycle. (b) Corresponding volumetric energy density and active capacity utilization of Se for Se-MSe electrodes. Active capacity utilization of Se is calculated as shown in part Calculation of Energy density.

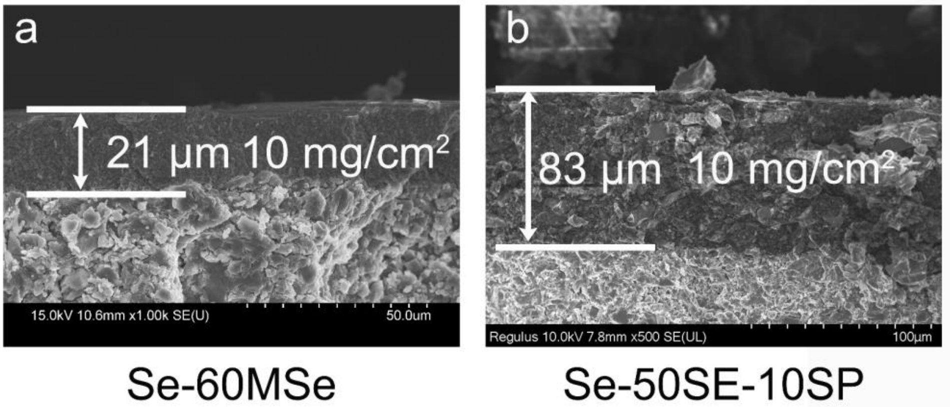

Fig. S9 | Cross-sectional SEM images of (a) Se-60MSe and (b) Se-50SE-10SP cathodes with the same mass loading of 10 mg/cm^2.

Table S4. Details for calculation of the density and porosity of Se-MSe composite cathodes in different proportions under different pressures.

	**Cathode**	**m_C [mg]**	**ρ_0 [g/cm^3]**	**Pressure/ MPa**			
				**0**	**100**	**270**	**360**
**h_C [mm]**				2.53	1.23	1.13	1.11
**ρ_C [g/cm^3]**	Se-10MSe	390	4.94	1.96	4.04	4.40	4.48
**φ_C [%]**				28.67	18.23	10.99	9.39
**h_C [mm]**				3.76	1.79	1.63	1.6
**ρ_C [g/cm^3]**	Se-20MSe	580	5.08	1.97	4.13	4.53	4.62
**φ_C [%]**				61.29	18.69	10.71	9.03
**h_C [mm]**				3.61	1.78	1.66	1.61
**ρ_C [g/cm^3]**	Se-30MSe	590	5.22	2.08	4.22	4.53	4.67
**φ_C [%]**				60.12	19.12	13.28	10.58
**h_C [mm]**				3.65	1.79	1.69	1.62
**ρ_C [g/cm^3]**	Se-40MSe	600	5.37	2.09	4.27	4.52	4.72
**φ_C [%]**				61.03	20.54	15.84	12.20
**h_C [mm]**				3.52	1.78	1.69	1.59
**ρ_C [g/cm^3]**	Se-50MSe	600	5.54	2.17	4.29	4.52	4.81
**φ_C [%]**				60.78	22.43	18.30	13.17
**h_C [mm]**				5.21	2.93	2.72	2.60
**ρ_C [g/cm^3]**	Se-60MSe	995	5.71	2.43	4.31	4.66	4.88
**φ_C [%]**				57.38	24.61	18.36	14.60
**h_C [mm]**				3.36	1.77	1.63	1.55
**ρ_C [g/cm^3]**	Se-70MSe	600	5.89	2.27	4.32	4.69	4.93
**φ_C [%]**				61.39	24.71	20.41	16.30
**h_C [mm]**				3.07	1.69	1.59	1.49
**ρ_C [g/cm^3]**	Se-80MSe	600	6.09	2.49	4.52	4.81	5.13
**φ_C [%]**				59.10	24.30	21.03	15.73
**h_C [mm]**				2.98	1.57	1.49	1.43
**ρ_C [g/cm^3]**	Se-90MSe	600	6.30	2.56	4.87	5.13	5.34
**φ_C [%]**				59.26	22.68	18.53	15.11

Table S5. Details for calculation of the density and porosity of Se-50SE-10SP composite cathodes.

	**Cathode**	**m_C [mg]**	**ρ_0 [g/cm^3]**	**Pressure/ MPa**			
				**0**	**100**	**270**	**360**
h_C [mm]				6.08	3.69	3.04	2.55
ρ_C [g/cm^3]	Se-SE-SP	300	2.31	0.63	1.04	1.26	1.50
φ_C [%]				72.8	54.9	45.5	35.1

Section 4. In situ observation of creep behavior of Se

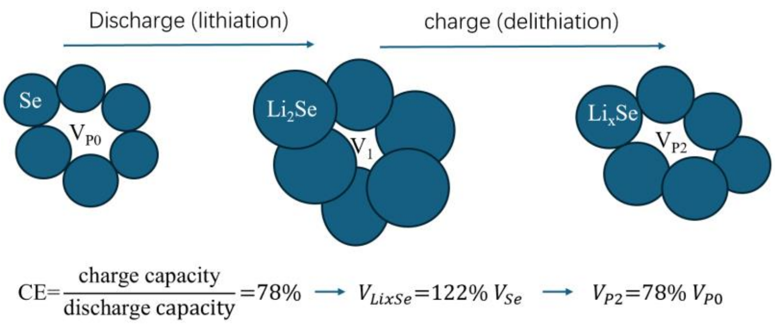

Fig. S10 | Schematic of the volume change of Se particles during (de)lithiation and the corresponding pore size.

The coulombic efficiency (CE) is obtained by discharge (lithiation) capacity divided by charge (delithiation) capacity. That is, if plastic deformation is not considered, after one cycle of charging and discharging, the degree of lithiation of Se is 22% compared to the initial state, i.e., the volume of Se increases by about 22% ($\Delta V_{Se/Li2Se}$=98%), approximating the relative shrinkage of the pores by 22%. This is to imply that at the end of charging, the pore size is approximated to be 78% of the initial state. Thus, the volume change of porosity can be used to correspond to the volume change to validate the creep mechanism, even though there will be minor variances in the local environment, and we present the change of 20 pores to emphasize static tendency.

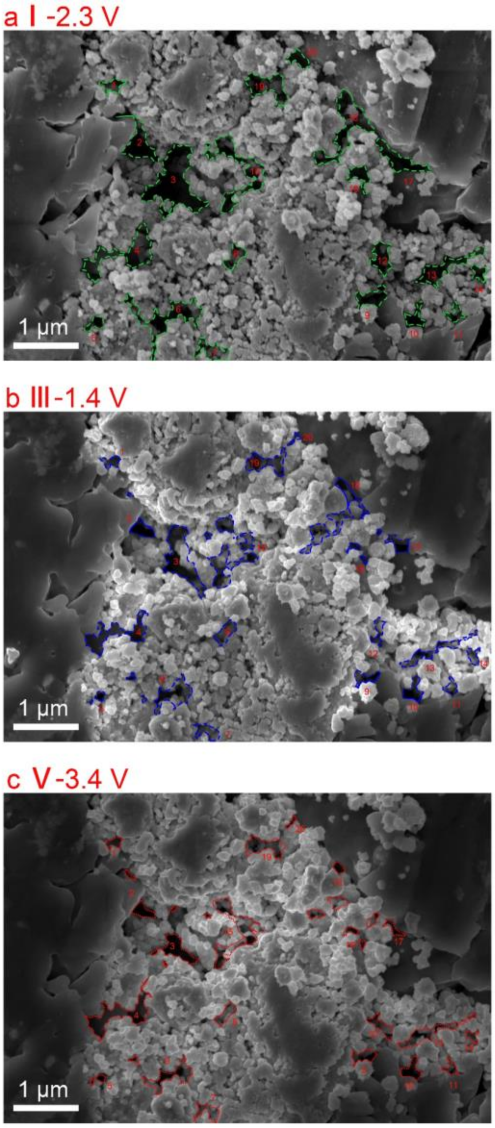

Fig. S11 | The morphology evolution of region I of the Se-60MSe cathode varies with different states of charge, corresponding to various creep changes of Se. The complete evolution in Movie S1.

We illustrate the evolution of the particle contacts by altering the pore sizes during charging and discharging process for the in-situ SEM observant ion. The area of the 20 pores in the initial condition of the Se-MSe cathode, after discharging to 1.4 V and after charging to 3.4 V are compared (the area data were obtained using histogram statistics from Photoshop).

If without creep deformation, the electrode pores theoretically shrink during discharge (lithiation) and re-expand to 78% of the volume of the initial state after end of charge (delithiation). However, most of the actual pore sizes after the end of charge are much smaller than 78% of the initial state (Fig. 3d in main text), indicating that Se creep into the pores to induce the pores further shrink.

Table S6. Material properties used to simulate the creep of composite electrodes.

Parameter (unit)	Value
Young's modulus of LCO[25] (Pa)	1.91×10^{11}
Young's modulus of Se (Pa)	1.8×10^{10}
Young's modulus of Li_2Se (Pa)	1.27×10^{10}
Young's modulus of MSe (Pa)	3.76×10^{10}
Young's modulus of Li_4MSe (Pa)	2.25×10^{10}
Poisson's ratio of LCO (1)	0.3
Poisson's ratio of Se (1)	0.3
Poisson's ratio of MSe (1)	0.3
Stress exponent of Se (Li_2Se) (1)	7 (4.9)
Creep rate coefficient of Se (1/s)	1×10^{3}
Reference stress of Se (Li_2Se) (Pa)	6.9×10^{9} (4.8×10^{9})

The Young's modulus of Se, Li_2Se, MSe, and Li_4MSe listed in Table S6 are obtained from the nanoindentation test in Fig. S22-S25, and the Young's modulus of LCO is from the previous reports[25].

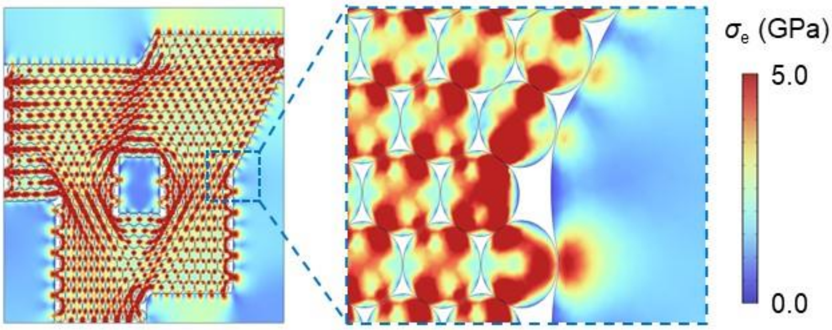

Fig. S12 | The deformation and equivalent stress in the fully lithiated LCO-MSe cathode.

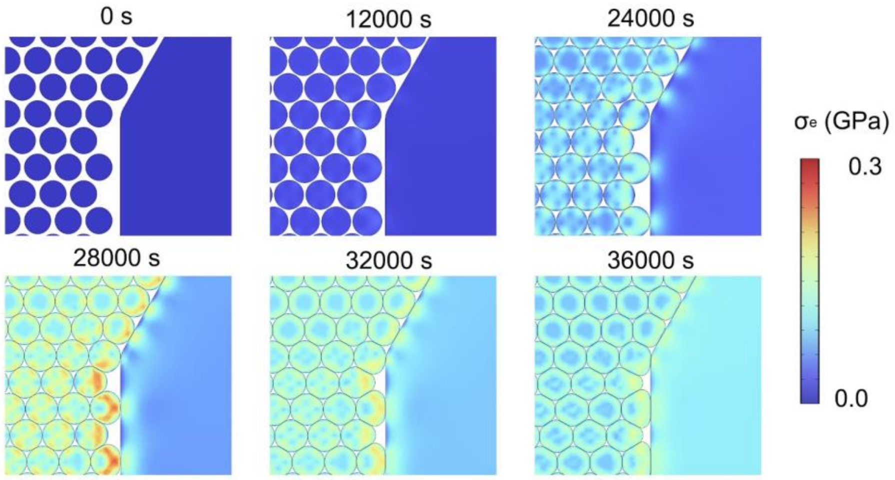

Fig. S13 | The equivalent stress in the Se-MSe cathode during its lithiation.

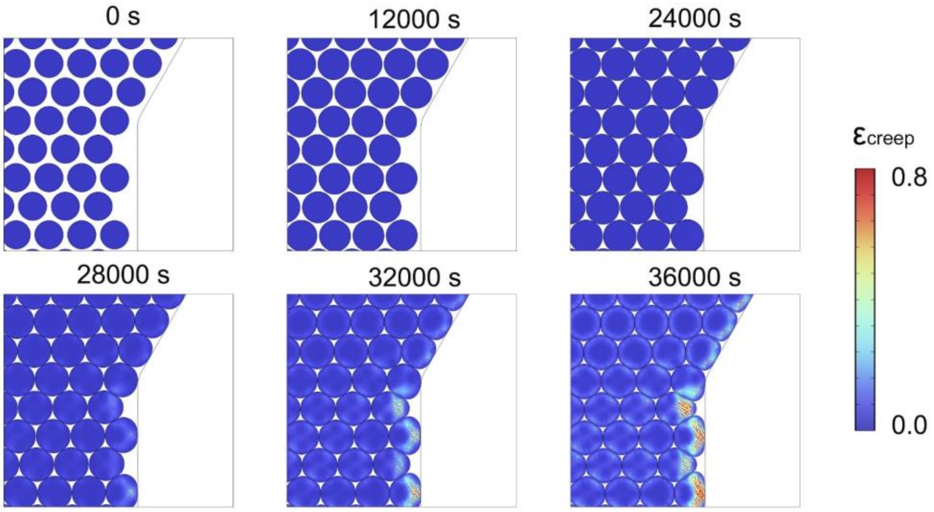

Fig. S14 | The magnitude (color contour) and direction (arrows) of the creep strain in the Se particles during the lithiation of the Se-MSe cathode.

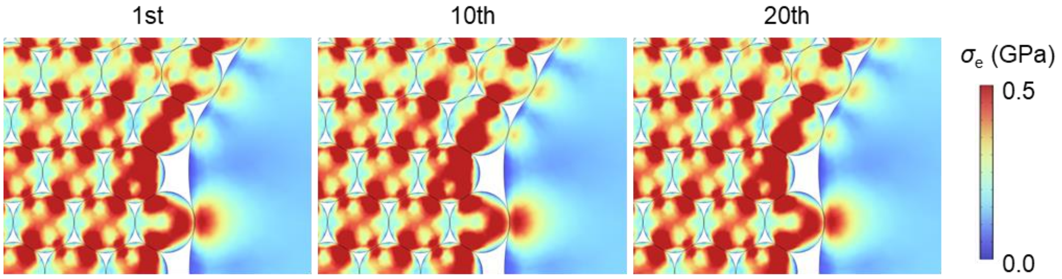

Fig. S15 | The deformation and equivalent stress in the LCO-MSe cathode at different cycles.

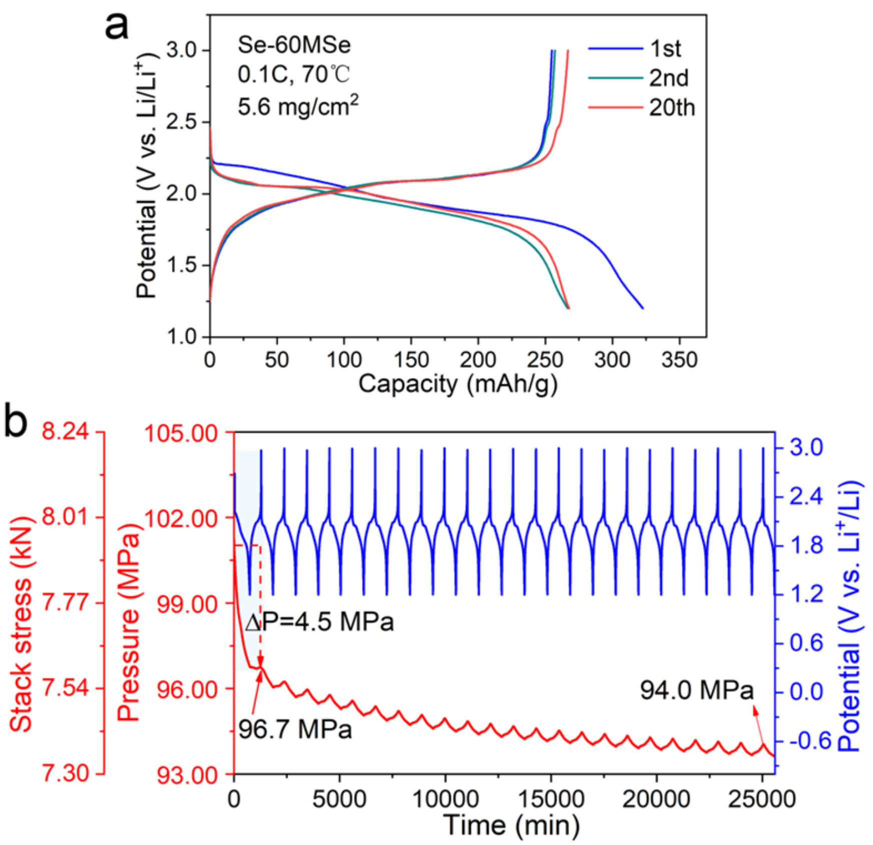

Fig. S16 | Operando pressure measurement for Se-60MSe|LPSC|InLi ASSB. (a) Galvanostatic curves of the battery. (b) Galvanostatic cycling of the battery along with the measured stack-pressure changes. The pressure at t = 0 is 101.2 MPa.

Section 5. Comparison for mechanical properties on creep behavior of materials

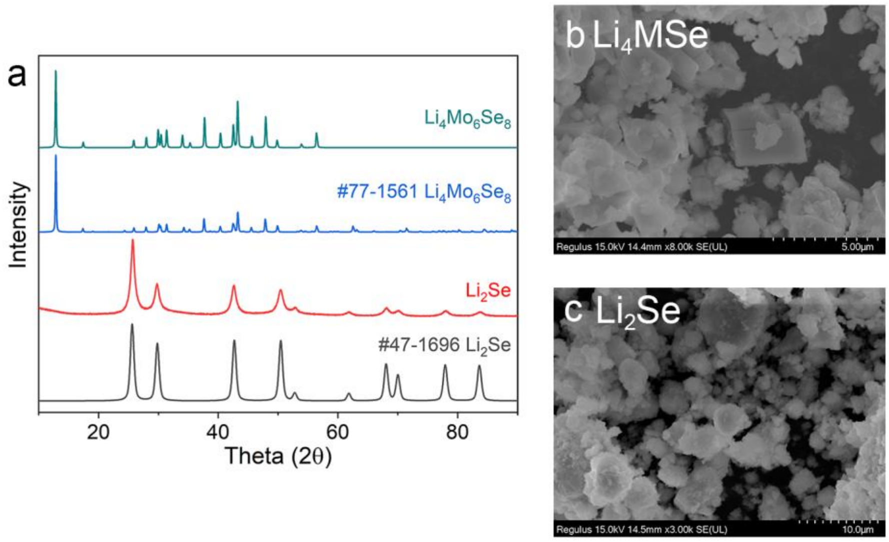

Fig. S17 | Structural and morphological characterization for Li_2Se and Li_4MSe obtained by chemical prelithiation. (a) XRD patterns of Li_2Se and Li_4MSe. SEM images of (b) Li_4MSe and (c) Li_2Se.

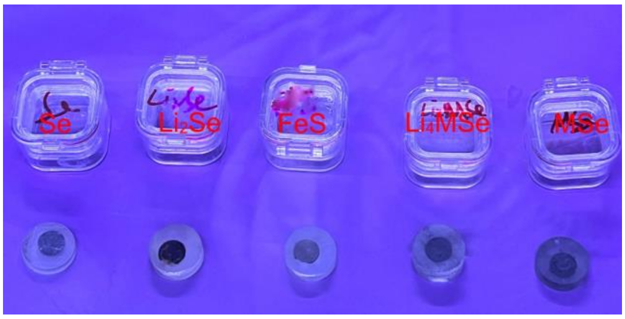

Fig. S18 | Digital photo of polished samples for nanoindentation. Black zone is sample, and transparent part is epoxy resin for cold inlay with sample fixed.

Li_2Se and Li_4MSe exhibit higher strain-rate sensitivity exponent and considerably lower hardness and Young's modulus after lithiation in comparison to Se and MSe (Fig. S21-S24). This tendency is consistent with earlier research on the development of Si hardness, elastic modulus, and strain-rate sensitivity exponent with lithiation degree[26].

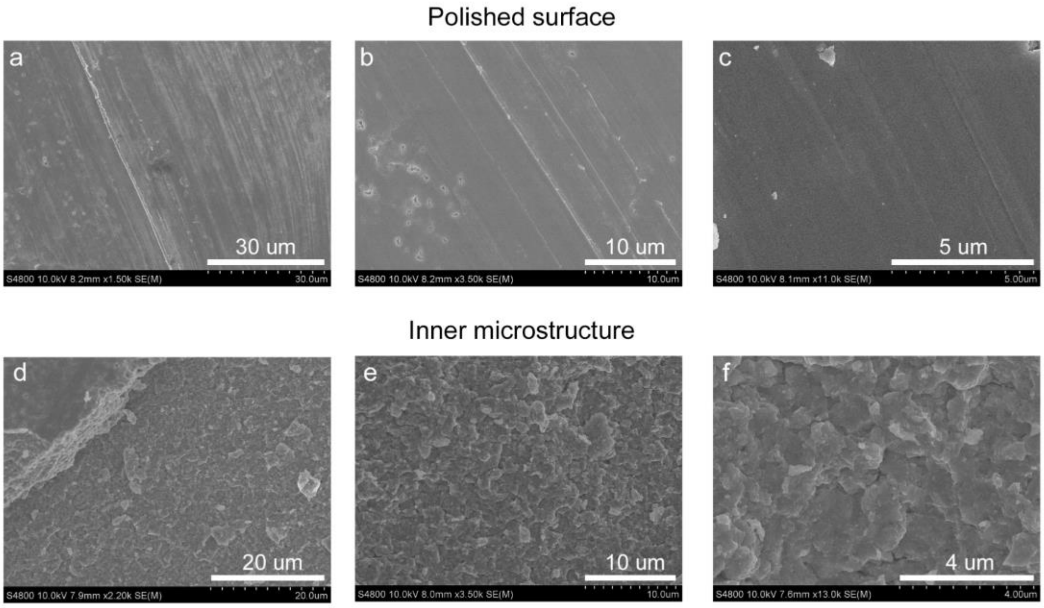

Fig. S19 | The surface morphology of cold-pressed Se samples used for nanoindentation. (a-c) The polished surface and (d-f) inner microstructure.

The Se pellet has a much dense contact in particles without invisible pores and cracks, consistent with its high relative density of 95.65% (measured by Archimedes method). The Young's modulus and hardness measured from such dense pellet should be close to the material's intrinsic properties.

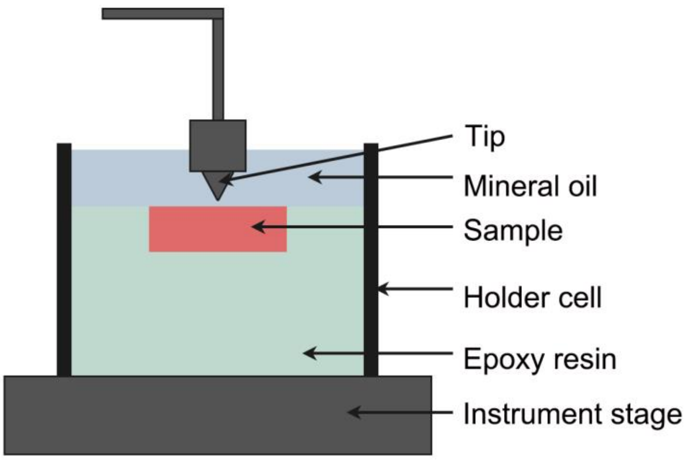

Fig. S20 | Schematic of specialized fluid cell with mineral oil to prevent the sulfide sample from being exposed to air during nanoindentation.

The schematic diagram's height of the liquid cell has been expanded for ease of understanding. In the actual device, the liquid bath is 1 mm high to protect the tip of the nanoindentation from being impacted. And the mineral oil is well anchored in the liquid cell due to its high surface tension.

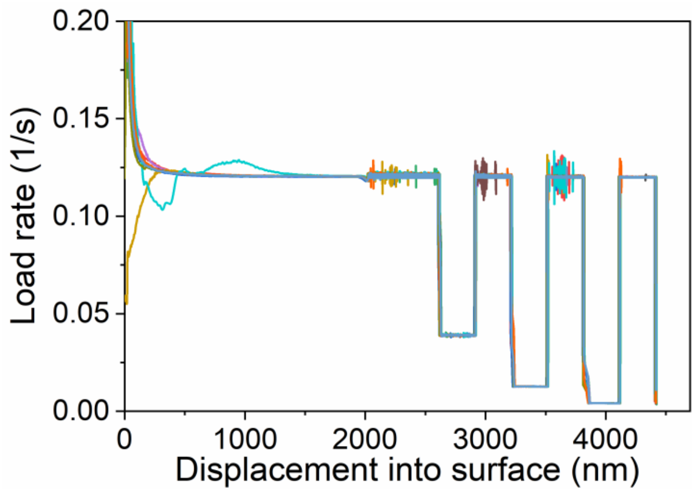

Fig. S21 | Nanoindentation strain-rate jump experiment by four different applied strain rates at corresponding displacement.

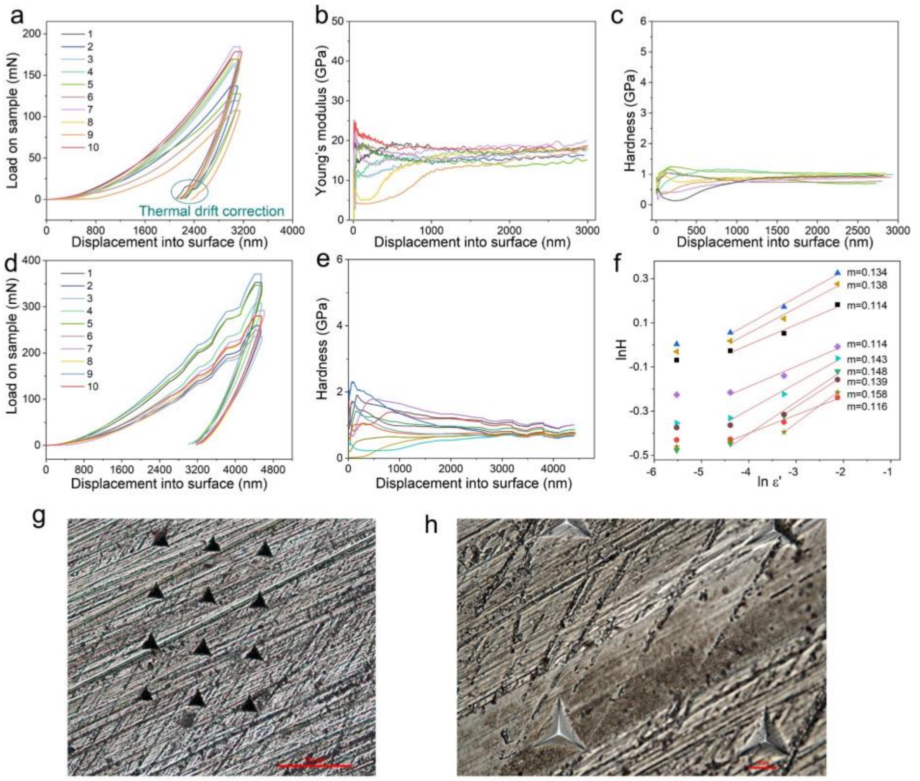

Fig. S22 | Mechanical characteristics of Se sample by nanoindentation test. (a-c) Displacement control mode for measurement of E and H by maintaining a constant strain rate of 0.05 s^{-1}. Using the Oliver-Pharr method, (a) the corresponding load–displacement curves and the calculated (b) E and (c) H. (d-f) Nanoindentation strain-rate jump experiment with dour different applied strain rates. (d) Corresponding load–displacement curves, (e) the measured H, and (f) the calculated strain-rate sensitivity exponent *m* for Se. (g, h) Residual topography after nanoindentation experiments by microscopy. Scale bar: 100 um for (g), 10 um for (h).

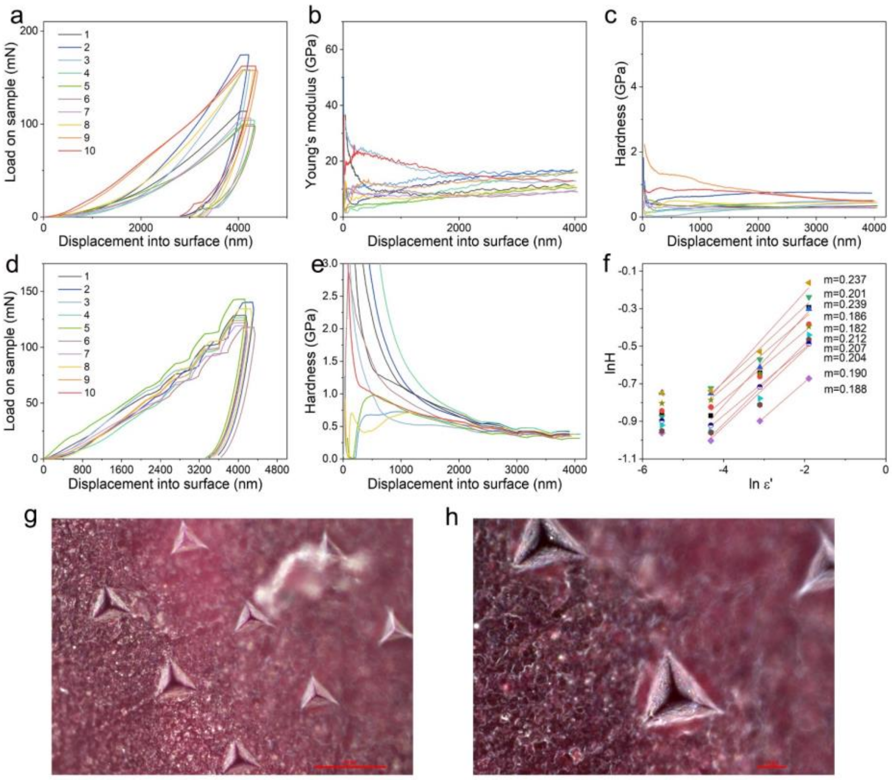

Fig. S23 | Mechanical characteristics of Li_2Se sample by nanoindentation test. (a-c) Displacement control mode for measurement of E and H by maintaining a constant strain rate of 0.05 s^{-1}. Using the Oliver-Pharr method, (a) the corresponding load–displacement curves and the calculated (b) E and (c) H. (d-f) Nanoindentation strain-rate jump experiment with dour different applied strain rates. (d) Corresponding load–displacement curves, (e) the measured H, and (f) the calculated strain-rate sensitivity exponent *m* for Li_2Se. (g, h) Residual topography after nanoindentation experiments by microscopy. Scale bar: 50 um for (g), 10 um for (h).

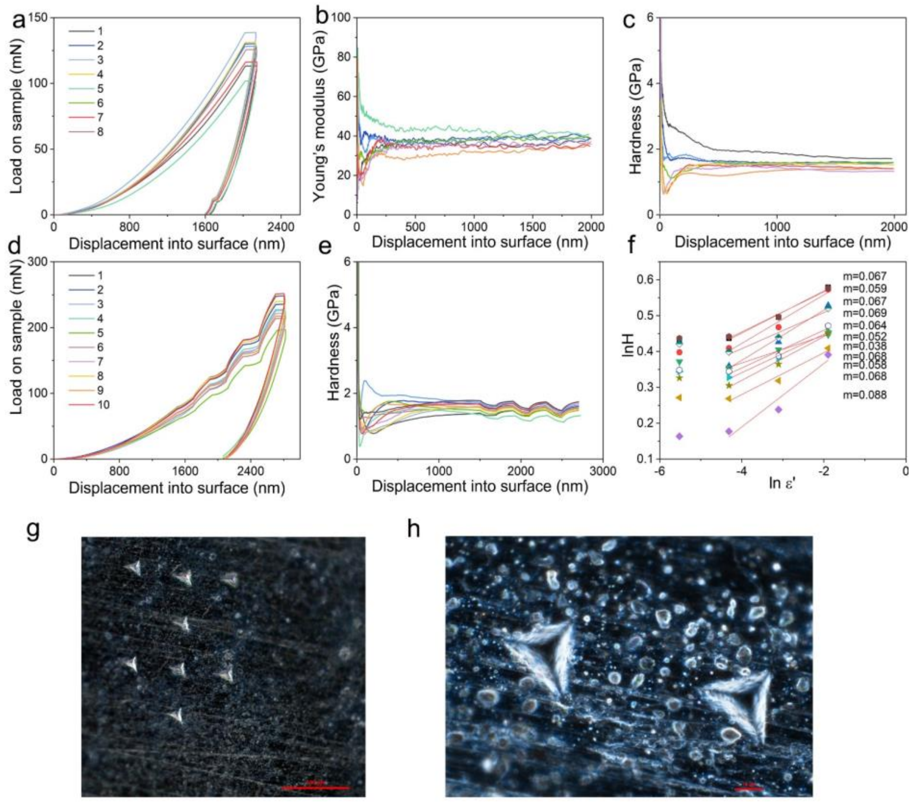

Fig. S24 | Mechanical characteristics of MSe sample by nanoindentation test. (a-c) Displacement control mode for measurement of E and H by maintaining a constant strain rate of 0.05 s^{-1}. Using the Oliver-Pharr method, (a) the corresponding load–displacement curves and the calculated (b) E and (c) H. (d-f) Nanoindentation strain-rate jump experiment with dour different applied strain rates. (d) Corresponding load–displacement curves, (e) the measured H, and (f) the calculated strain-rate sensitivity exponent *m* for MSe. (g, h) Residual topography after nanoindentation experiments by microscopy. Scale bar: 100 um for (g), 10 um for (h).

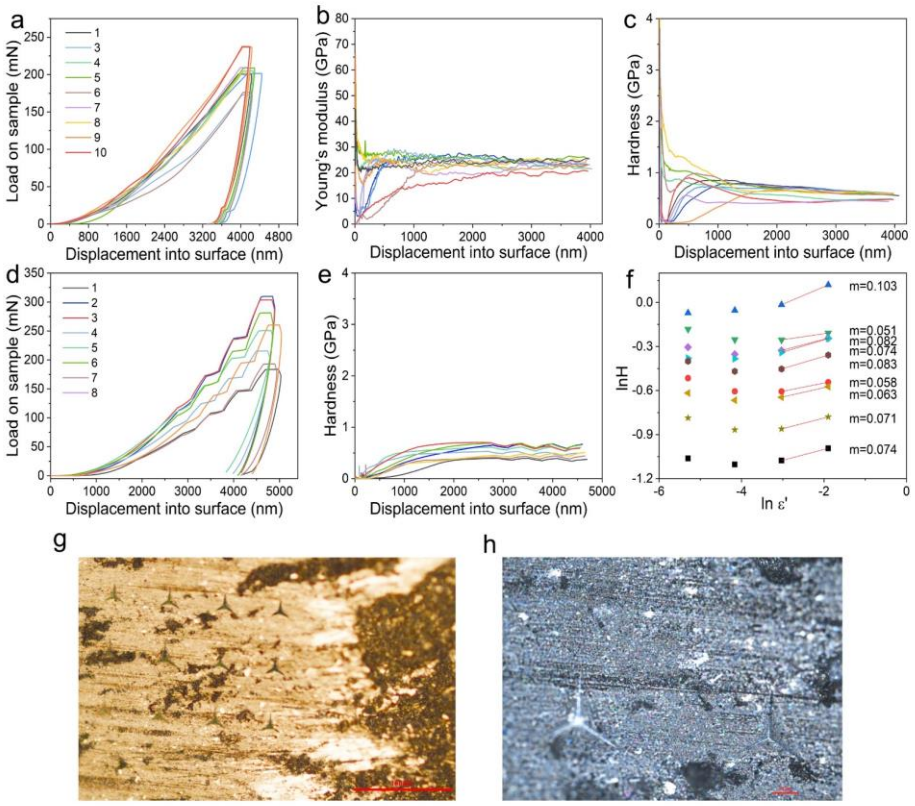

Fig. S25 | Mechanical characteristics of Li_4MSe sample by nanoindentation test. (a-c) Displacement control mode for measurement of E and H by maintaining a constant strain rate of 0.05 s^{-1}. Using the Oliver-Pharr method, (a) the corresponding load–displacement curves and the calculated (b) E and (c) H. (d-f) Nanoindentation strain-rate jump experiment with dour different applied strain rates. (d) Corresponding load–displacement curves, (e) the measured H, and (f) the calculated strain-rate sensitivity exponent *m* for Li_4MSe. (g, h) Residual topography after nanoindentation experiments by microscopy. Scale bar: 100 um for (g), 10 um for (h).

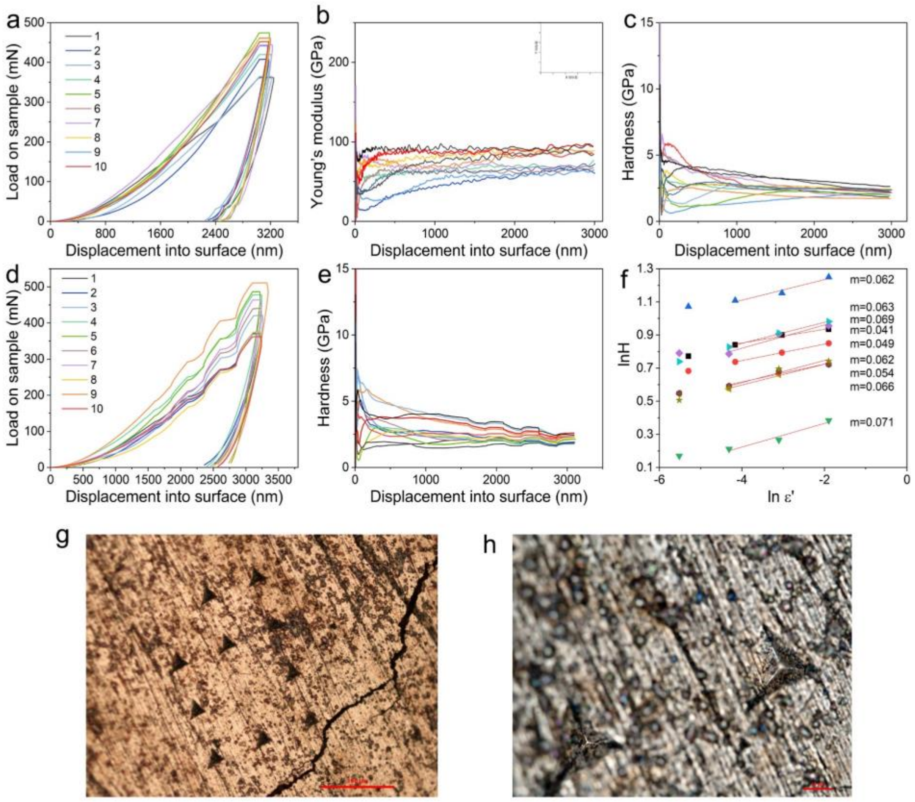

Fig. S26 | Mechanical characteristics of FeS sample by nanoindentation test. (a-c) Displacement control mode for measurement of E and H by maintaining a constant strain rate of 0.05 s^{-1}. Using the Oliver-Pharr method, (a) the corresponding load–displacement curves and the calculated (b) E and (c) H. (d-f) Nanoindentation strain-rate jump experiment with dour different applied strain rates. (d) Corresponding load–displacement curves, (e) the measured H, and (f) the calculated strain-rate sensitivity exponent *m* for FeS. (g, h) Residual topography after nanoindentation experiments by microscopy. Scale bar: 100 um for (g), 10 um for (h).

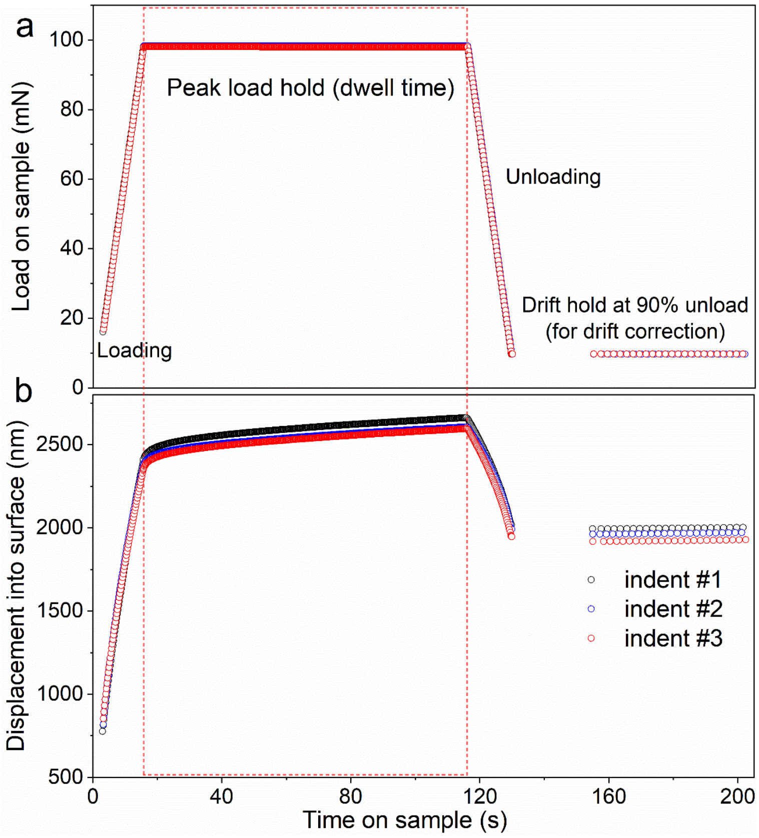

Fig. S27 | The constant load and hold method for creep measurements of Se material. The loading rate is 6.67 mN/s and the holding times is 100 s. (a) The load on the sample. (b) The displacement into surface curves versus time on the sample. This indentation depth trend with time is similar to the creep evolution on constant stress in previous research[27].

As illustrated in this constant load test by nanoindentation, the indentation depth increases continuously during the long-term dwell time, indicating the viscosity feature of Se material.

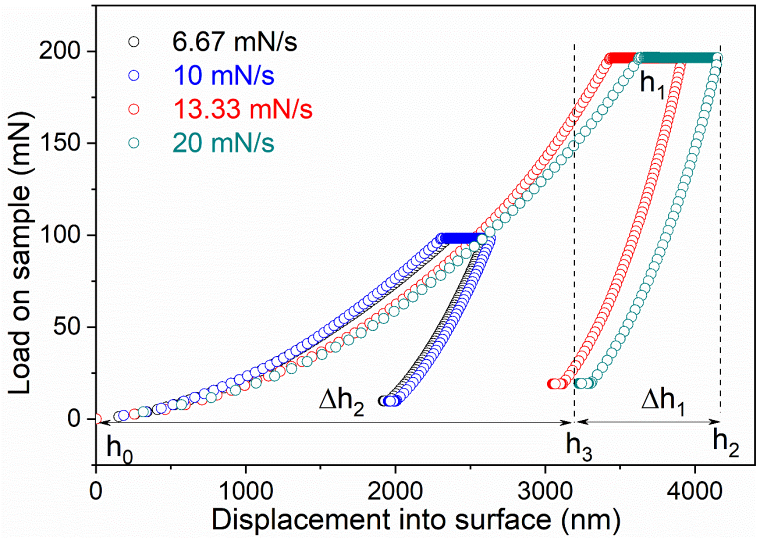

Fig. S28 | Load-displacement curves of Se sample at different loading rates and maximum loads (hold at the max load for 100 s).

After unloading, the indentation depth reverts from h_2 to h_3 (Δh_1), leaving the non-reversible part (Δh_2) due to the plastic deformation, which well further demonstrates that the Se material exhibits viscoplasticity.

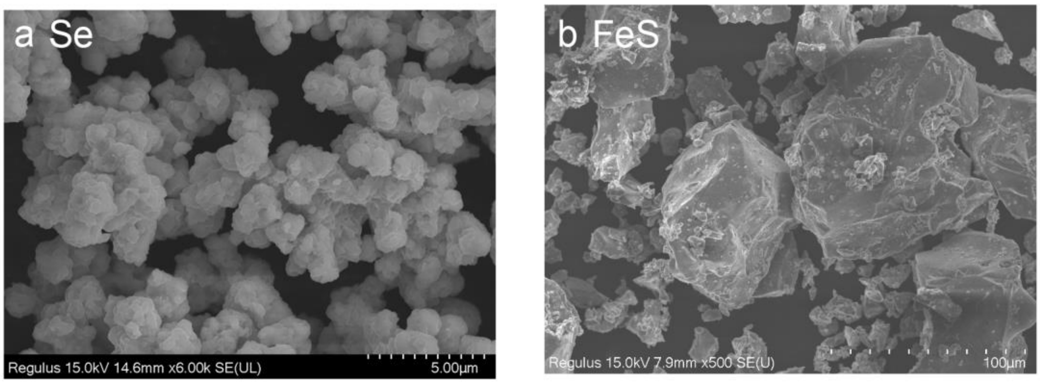

Fig. S29 | Comparison of particle size and morphology. (a) Se, (b) FeS.

The morphology and size of the Se and FeS particles vary greatly, despite their similar densities (Se, 4.81 g/cm^3; FeS, 4.84 g/cm^3), which may have led to the FeS-MSe electrode being thicker than the Se-MSe electrode when they have the same mass loadings.

Section 6. Electrochemical performance of creep-type Se-60MSe cathode.

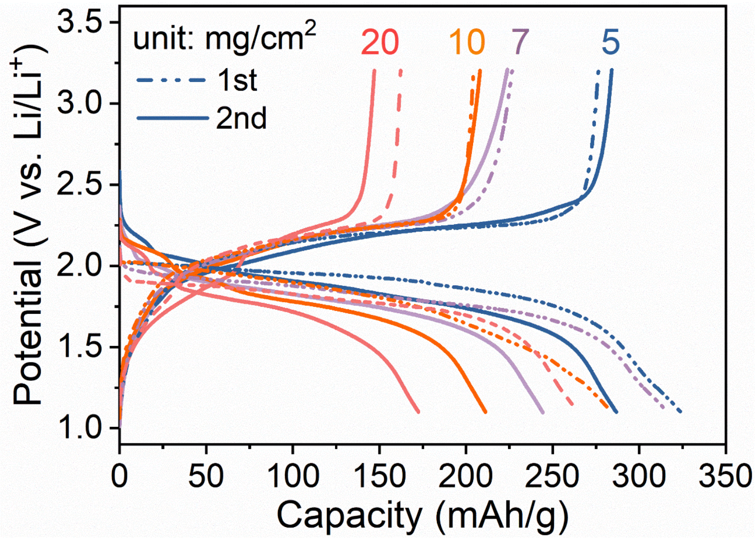

Fig. S30 | The galvanostatic curves of Se-60MSe cathodes with varying loadings.

There are more electrochemical reaction sites in the two-phase cathode and where ions and electrons migrating in the same medium in Se-MSe cathodes significantly reduce the tortuosity factor,[28] benefiting the cathode kinetics with an excellent performance even at high mass loadings and great current densities.

Table S7. Calculation details of the energy density of Se-60MSe cathode with different mass loadings.

Mass Loading [mg/cm^2]	Cycle	Specific Capacity [mAh/g]	Capacity [mAh/cm^2]	$V_{average}$ [V]	$h_{cathode}$ [um]	E_G [Wh/kg]	E_V [Wh/L]
3	1st	323.4	0.97	2.08	8.2	688.8	**2460**
	2nd	283.5	0.85	2.10	8.2	609.5	**2178**
5	1st	321.9	1.61	2.04	13.5	672.8	**2432**
	2nd	271.9	1.36	2.03	13.5	565.6	**2044**
10	1st	286	2.86	1.97	21	569.3	**2505**
	2nd	211.4	2.11	1.94	21	410.1	**1949**

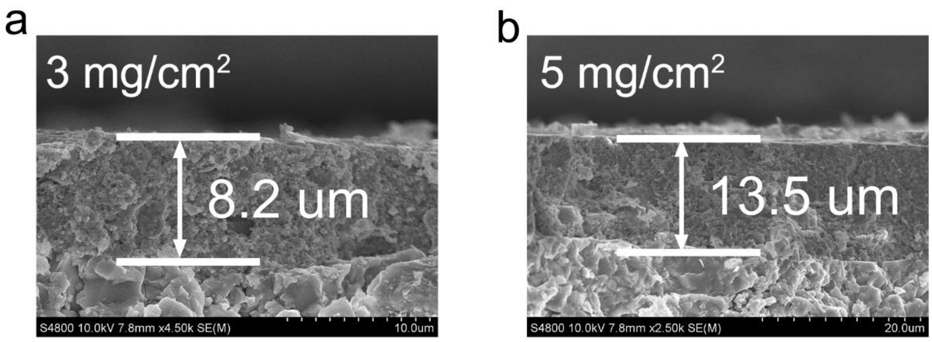

Fig. S31 | The thickness of Se-60MSe cathode with different mass loadings. (a) 3 mg/cm^2, (b) 5 mg/cm^2.

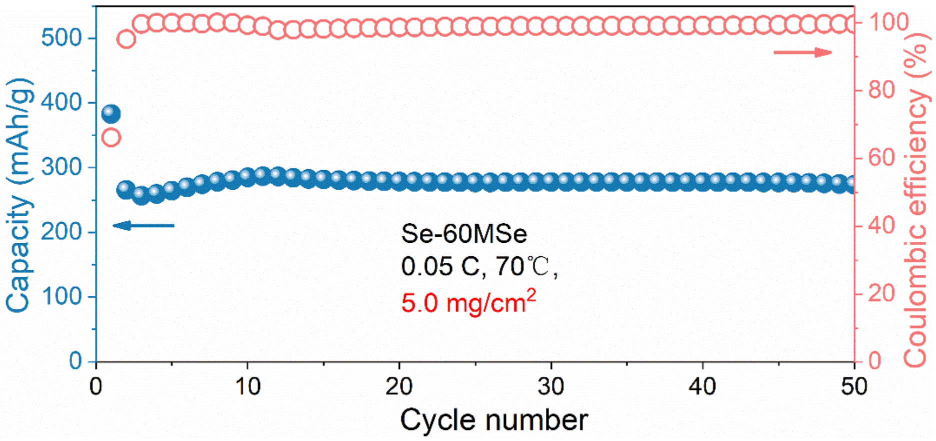

Fig. S32 | Cycling performance of Se-60MSe cathode with the mass loading of 5 mg/cm^2.

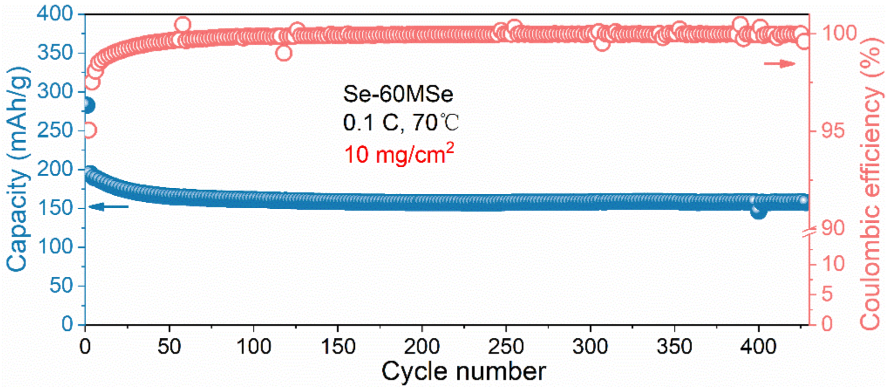

Fig. S33 | Cycling performance of Se-60MSe cathode with the mass loading of 10 mg/cm^2.

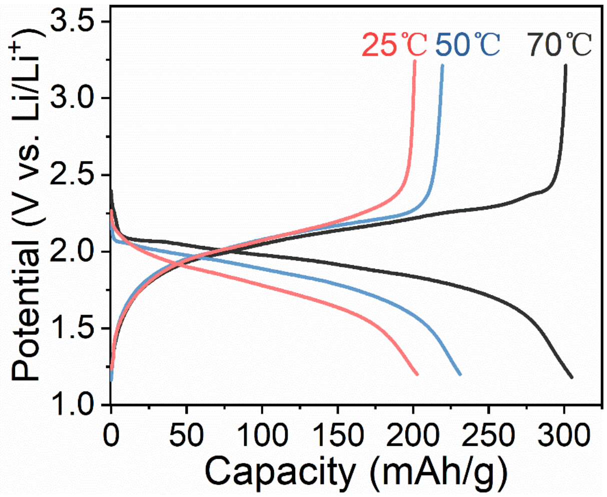

Fig. S34 | The 2nd galvanostatic curves of Se-60MSe cathodes at different temperatures.

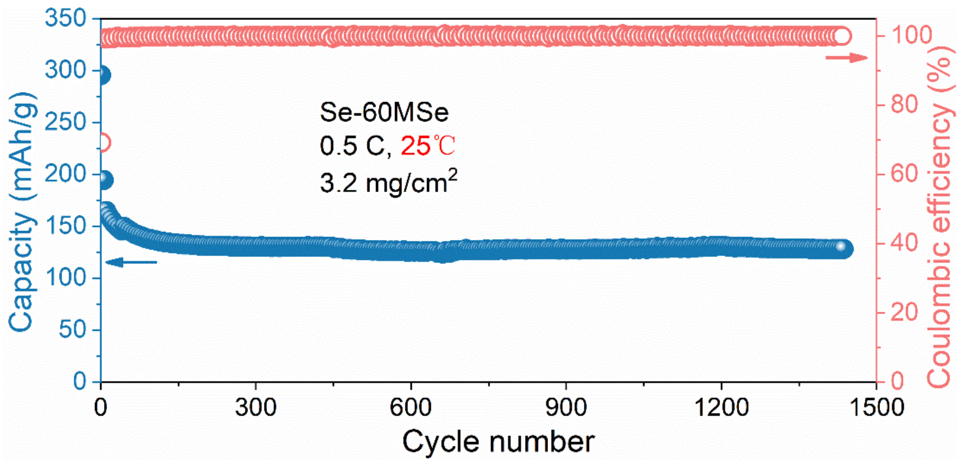

Fig. S35 | Cycling performance of Se-60MSe cathode at room temperature of 25℃ at 0.5 C.

Table S8. Calculation details of the energy density of Se-60MSe cathode for the CT-ASS Se-60MSe//InLi all-solid-state pouch battery.

	**Parameter**	**Value**
**Se-60MSe cathode (at the cathode level)**	Area weight	4.69 mg/cm^2
	Area capacity	1.66 mAh/cm^2
	Thickness	18 μm
	Average voltage (vs InLi)	1.299 V
	Average voltage (vs Li/Li$^+$)	1.899 V
	Capacity	26.53 mAh
	Weight	76.53 mg
	Energy density	658.3 Wh/kg
		1749.3 Wh/L

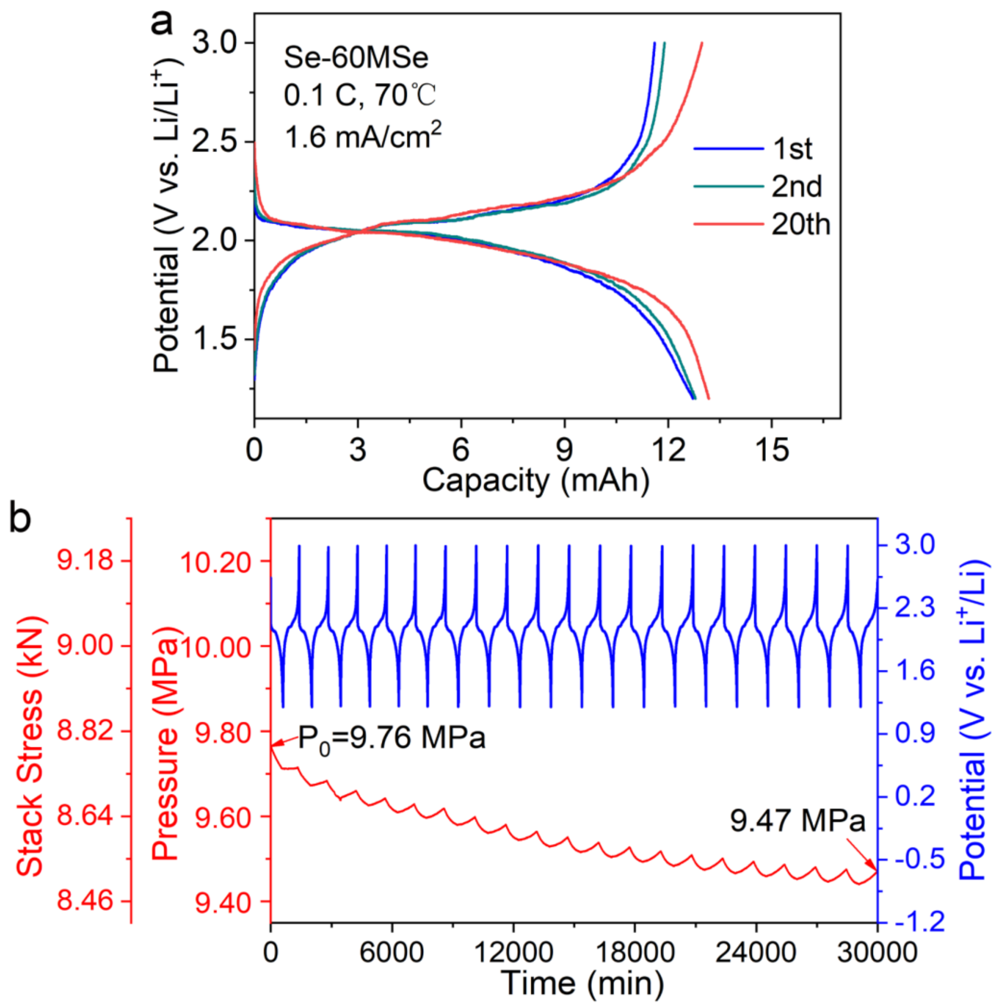

Fig. S36 | Operando pressure measurement for Se-60MSe|LPSC|InLi (3 cm×3 cm, 1.48 mAh/cm^2) all-solid-state pouch cell. (a) Galvanostatic curves of the battery. (b) Galvanostatic cycling of the battery along with the measured stack-pressure changes. The pressure at t = 0 is 9.76 MPa.

References

1 Berg, E. J., Villevieille, C., Streich, D., Trabesinger, S. & Novák, P. Rechargeable Batteries: Grasping for the Limits of Chemistry. *Journal of The Electrochemical Society* **162**, A2468-A2475 (2015). https://doi.org:10.1149/2.0081514jes

2 Li, M. *et al.* Dense All-Electrochem-Active Electrodes for All-Solid-State Lithium Batteries. *Advanced Materials* **33** (2021). https://doi.org:10.1002/adma.202008723

3 Aspinall, J. *et al.* Effect of Microstructure on the Cycling Behavior of Li–In Alloy Anodes for Solid-State Batteries. *ACS Energy Letters* **9**, 578-585 (2024). https://doi.org:10.1021/acsenergylett.3c02274

4 Huang, T. S. & Brittain, J. O. Defect structure and mechanical behavior of B LiIn. *Materials Science and Engineering* **93**, 83-92 (1987). https://doi.org:https://doi.org/10.1016/0025-5416(87)90414-9

5 Mallakpour, F., Kasraie, M., Herbert, E. G., Phani, P. S. & Hackney, S. A. Length-scale-dependent stress relief mechanisms in indium at high homologous temperatures. *Journal of Materials Research* **36**, 2444-2455 (2021). https://doi.org:10.1557/s43578-021-00186-6

6 Fathiannasab, H., Zhu, L. & Chen, Z. Chemo-mechanical modeling of stress evolution in all-solid-state lithium-ion batteries using synchrotron transmission X-ray microscopy tomography. *Journal of Power Sources* **483** (2021). https://doi.org:10.1016/j.jpowsour.2020.229028

7 Tian, H.-K., Chakraborty, A., Talin, A. A., Eisenlohr, P. & Qi, Y. Evaluation of The Electrochemo-Mechanically Induced Stress in All-Solid-State Li-Ion Batteries. *Journal of The Electrochemical Society* **167** (2020). https://doi.org:10.1149/1945-7111/ab8f5b

8 Jankovský, O., Kovařík, J., Leitner, J., Růžička, K. & Sedmidubský, D. Thermodynamic properties of stoichiometric lithium cobaltite LiCoO2. *Thermochimica Acta* **634**, 26-30 (2016). https://doi.org:https://doi.org/10.1016/j.tca.2016.04.018

9 Gauthier, M. *et al.* Melt Casting LiFePO4 : I. Synthesis and Characterization. *Journal of The Electrochemical Society* **157**, A453 (2010). https://doi.org:10.1149/1.3284505

10 Deaton, B. C. & Blum, F. A. Properties of Group VI B Elements Under Pressure. I. Melting Curves of S, Se, and Te. *Physical Review* **137**, A1131-A1138 (1965). https://doi.org:10.1103/PhysRev.137.A1131

11 Soma, T. & Matsuo, H. Pressure derivatives of the melting point for Si and Ge. *Journal of Physics C: Solid State Physics* **15**, 1873 (1982). https://doi.org:10.1088/0022-3719/15/9/010

12 Wang, F.-q. *et al.* Effects of low melting point metals (Ga, In, Sn) on hydrolysis properties of aluminum alloys. *Transactions of Nonferrous Metals Society of China* **26**, 152-159 (2016). https://doi.org:https://doi.org/10.1016/S1003-6326(16)64100-6

13 Geng, H., Zhang, G., Wang, Z., Deng, Y. & Qin, H. Density–temperature properties of Ga–Sb alloy melt. *Applied Physics A* **98**, 227 (2009). https://doi.org:10.1007/s00339-009-5380-2

14 Koerver, R. *et al.* Chemo-mechanical expansion of lithium electrode materials – on the route to mechanically optimized all-solid-state batteries. *Energy & Environmental Science* **11**, 2142-2158 (2018). https://doi.org:10.1039/c8ee00907d

15 Nitta, N., Wu, F., Lee, J. T. & Yushin, G. Li-ion battery materials: present and future. *Materials Today* **18**, 252-264 (2015). https://doi.org:10.1016/j.mattod.2014.10.040

16 Van der Ven, A., Marianetti, C., Morgan, D. & Ceder, G. Phase transformations and volume changes in spinel LixMn2O4. *Solid State Ionics* **135**, 21-32 (2000). https://doi.org:https://doi.org/10.1016/S0167-2738(00)00326-X

17 Wu, F., Maier, J. & Yu, Y. Guidelines and trends for next-generation rechargeable lithium and lithium-ion batteries. *Chem Soc Rev* **49**, 1569-1614 (2020). https://doi.org:10.1039/c7cs00863e

18 Zhang, B.-W. *et al.* Gallium-based liquid metals for lithium-ion batteries. *Interdisciplinary Materials* **1**, 354-372 (2022). https://doi.org:https://doi.org/10.1002/idm2.12042

19 Wu, F. & Yushin, G. Conversion cathodes for rechargeable lithium and lithium-ion batteries. *Energy & Environmental Science* **10**, 435-459 (2017). https://doi.org:10.1039/c6ee02326f

20 Chang, D. *et al.* Elucidating the origins of phase transformation hysteresis during electrochemical cycling of Li–Sb electrodes. *Journal of Materials Chemistry A* **3**, 18928-18943 (2015). https://doi.org:10.1039/C5TA06183K

21 Santhosha, A. L., Medenbach, L., Buchheim, J. R. & Adelhelm, P. The Indium−Lithium Electrode in Solid‐State Lithium‐Ion Batteries: Phase Formation, Redox Potentials, and Interface Stability. *Batteries & Supercaps* **2**, 524-529 (2019). https://doi.org:10.1002/batt.201800149

22 Coleman, S. T., McKinnon, W. R. & Dahn, J. R. Lithium intercalation inLixMo6Se8: A model mean-field lattice gas. *Physical Review B* **29**, 4147-4149 (1984). https://doi.org:10.1103/PhysRevB.29.4147

23 Yang, X., Li, X., Adair, K., Zhang, H. & Sun, X. Structural Design of Lithium–Sulfur Batteries: From Fundamental Research to Practical Application. *Electrochemical Energy Reviews* **1**, 239-293 (2018).

https://doi.org:10.1007/s41918-018-0010-3

24 Huang, X. *et al.* Advanced High-Performance Potassium–Chalcogen (S, Se, Te) Batteries. *Small* **17**, 2004369 (2021). https://doi.org:https://doi.org/10.1002/smll.202004369

25 Cheng, E. J., Taylor, N. J., Wolfenstine, J. & Sakamoto, J. Elastic properties of lithium cobalt oxide (LiCoO2). *Journal of Asian Ceramic Societies* **5**, 113-117 (2017). https://doi.org:https://doi.org/10.1016/j.jascer.2017.03.001

26 de Vasconcelos, L. S., Xu, R. & Zhao, K. Operando Nanoindentation: A New Platform to Measure the Mechanical Properties of Electrodes during Electrochemical Reactions. *Journal of The Electrochemical Society* **164**, A3840 (2017). https://doi.org:10.1149/2.1411714jes

27 Blum, W., Eisenlohr, P. & Breutinger, F. Understanding creep—a review. *Metallurgical and Materials Transactions A* **33**, 291-303 (2002). https://doi.org:10.1007/s11661-002-0090-9

28 Li, M. *et al.* All-in-One Ionic–Electronic Dual-Carrier Conducting Framework Thickening All-Solid-State Electrode. *ACS Energy Letters* **7**, 766-772 (2022). https://doi.org:10.1021/acsenergylett.1c02666
