# Supplementary material for: Creep-type all-solid-state cathode achieving long life
Source: Nat Commun. 2024 May 2;15:3706. doi: 10.1038/s41467-024-48174-8 (PMC11065878; doi:10.1038/s41467-024-48174-8)
Supplement: Supplementary file 3 — Description of Additional Supplementary Files [file 41467_2024_48174_MOESM3_ESM.pdf]

Description of Additional Supplementary Files

Supplementary Movie 1: In-situ observation for morphology evolution of Se-60MSe cathode during galvanostatic discharging and charging.
